# Notes and News

**Published:** 1896-12-12

**Authors:** 


### Notes and News.

(Collected and Communicated.)

The latest addition to the University College, Liverpool, is the Gossage Chemical Laboratory, which is to be opened by the Earl of Derby on the 12th inst.

A new ward for cancer patients is to be erected in connection with Friedenheim, the Home of Peace for the Dying at Swiss Cottage.

Dr. Blacker has been appointed to take charge, under Dr. Turney, of the Röntgen Rays photographic branch in connection with the electrical department of St. Thomas's Hospital.

The Director-General, Army Medical Department, draws our attention to the fact that a competitive examination for thirty-five commissions in the Army Medical Staff will be held on February 5th next and following days.

We learn that a scheme has been started for establishing in Southport a hospital for diseases of the eye, throat, and ear. Would it not have cost just as little initially to construct a department in connection with the hospital for the treatment of diseases of these organs? Certainly the administrative expense would have been infinitely less.

Dr. Cullingworth's medical friends and sympathisers are subscribing a sum to defray his expenses in the recent legal action. A sum of £500, we believe was received within the first forty-eight hours of the opening of the subscription list, and during the week more than enough has been received to cover everything. We heartily congratulate Dr. Cullingworth and those charged with the duty of collecting upon this splendid result.

The good people of Manchester are now awaking to the fact that £70,000 is a large sum to slip through their fingers; and, although it has been announced that the generous offer is definitely withdrawn, one more attempt is to be made to secure its benefits. We learn that a conference is to be arranged between the Mayors of Manchester and Salford, the trustees of the David Lewis Trust, and representatives of the two hospitals in question, with a view of talking the matter over once more.

At a special meeting of the Committee of the Royal Free Hospital, Gray's Inn Road, held on Friday, the 4th inst., Mr. Alfred Cock, Q.C., was unanimously elected a member of that body. Miss Julia Cock, M.D., Mr. Cock's sister, and Sub-Dean of the London School of Medicine for Women, has been on the committee for some time. Included in the other important business dealt with at the meeting was the consideration and adoption of the recommendation of the Weekly Board, to supply all the in-patients with tea, sugar, and butter. Hitherto, the in-patients, except the poorest of them, have had to provide these articles themselves.

Mr. Treloar is appealing to the public for funds, by aid of which the Christmas feast given at the Guildhall to 1,500 poor children, selected by the Ragged School Union from among the poorest districts in London, may be supplemented by hampers for the sick and crippled brothers and sisters of these children, who on account of their infirmities would otherwise be debarred from any share in the good things. It is a worthy object, and we commend it to our readers. No one will be the worse, and many a man will be far the better, for knowing, while he eats his Christmas dinner, that in some dark alley or in some little tenement, which but for his charity would be cold and comfortless, a cripple child is acting host, that a little child feast is going on, that empty stomachs are being filled and careworn faces made to smile, and that a mother is thinking kindly of the giver of the hamper.

At the annual meeting of the Belfast Royal Hospital a strong appeal was made to the governors to increase the hospital accommodation. Professor Whitla, in moving the adoption of the medical report, drew a comparison between the number of beds available in Belfast and in other towns, from which it appeared that, excluding the poor law wards, while Belfast had but 470 hospital beds, including all the special institutions, Edinburgh had 800 beds in one hospital, and 200 more in other institutions, Glasgow had 2,700 beds, and Dublin, with a smaller population than Belfast, had 2,500 beds. The feeling seems to be growing that the long reign of Queen Victoria should be commemorated by the erection of a hospital, in some degree commensurate with the growing importance of the city, and at the quarterly meeting of the general committee of the hospital the urgent necessity of building a new and complete hospital on a new site was warmly supported by Dr. Cuming.

Some dissatisfaction is felt at Cambridge that the new improvements in the operating theatre at the Addenbrooke's Hospital are to so far exceed the originally estimated sum. In August of the present year a provisional estimate of £1,000 was mentioned, which the president of Addenbrooke's most generously presented to the institution himself. Now that the plans have been gone into in detail it has been found that another £750 will be necessary to render the theatre complete and efficient in every respect. This sum is now asked to be provided from the reserve fund of the hospital, from which it was originally intended to draw the sum of £1,000, which the gift of the president rendered unnecessary. It is a pity that the original estimate was not sufficiently large to cover all contingencies, but in face of the opinion of so experienced an architect as Mr. Keith D. Young it is hardly likely that the hospital authorities will refuse to expend money which is regarded as entirely necessary for the efficiency of the institution and the well-being of the patients.

